# Young people’s sense of agency and responsibility towards promoting mental health in Brazil: a reflexive thematic analysis

**DOI:** 10.1136/bmjopen-2024-084996

**Published:** 2024-12-07

**Authors:** Josimar Antônio de Alcântara Mendes, Sheila Giardini Murta, Felipe Rodrigues Siston, Rafaela de Oliveira da Cunha, Brenda Thallys Rocha Seabra, Julyana Alves Ferreira, Rafa Ribeiro Alves de Souza, Victor Hugo de Lima Santos, Ilina Singh, Gabriela Pavarini

**Affiliations:** 1 Institute of Psychology, University of Brasilia, Brasília, Brazil; 2 Responsible Technology Institute, Department of Computer Science, University of Oxford, Oxford, UK; 3 Engajadamente Youth Collaborative Group, Brasília, UK; 4 Department of Psychiatry, University of Oxford, Oxford, UK; 5 Department of Social Policy and Intervention, University of Oxford, Oxford, UK; 6 Ethox Centre, Oxford Population Health, University of Oxford, Oxford, UK

**Keywords:** MENTAL HEALTH, Child & adolescent psychiatry, Community child health, Patient Participation, Psychosocial Intervention, Social Support

## Abstract

**Abstract:**

**Objectives:**

This study investigated how Brazilian young people perceive their role in promoting and supporting their peer community’s mental health and well-being, and the conditions and contexts influencing their engagement.

**Design:**

Co-produced qualitative study using in-depth interviews and focus groups with adolescents. The sessions were audio-recorded, transcribed and anonymised. Data were analysed using reflexive thematic analysis.

**Setting:**

Data collection took place remotely across Brazil via audio or video calls conducted by a youth collaborator and a senior researcher.

**Participants:**

46 Brazilian adolescents aged between 15 and 18 years old, recruited primarily via social media.

**Results:**

Our analysis generated four overarching themes: (1) young people’s sense of responsibility and motivation—many participants felt committed to promoting the mental health and well-being of their friends and community, while also critically emphasising responsibilities of and partnerships with adult stakeholders to make a meaningful difference; (2) strategies for promoting peer mental health—suggested strategies included peer-to-peer support, such as non-judgemental listening, and collective actions such as forming school groups; (3) intrapersonal barriers—key barriers to participation included a lack of necessary skills and self-efficacy; and (4) contextual barriers—young people reported feeling unheard, invalidated, and fearing judgement due to mental health stigma.

**Conclusion:**

These findings support an ecological view of youth participation in the promotion of mental health as dependent on intrapersonal, interpersonal and contextual factors. Realising young people’s potential in advancing mental health in their communities requires fostering youth–adult partnerships, providing peer support training, and combating adultism and mental health stigma.

**STRENGTHS AND LIMITATIONS OF THIS STUDY**

- A Youth Collaborative Group co-produced the study across all stages, helping shape the questions and methods to young people’s priorities and interests.
- Data were collected via youth-facilitated focus groups and interviews with Brazilian young people across the five Brazilian macro-regions.
- The sample is potentially biased towards those motivated to take part in mental health initiatives and who had the means to participate in a remote interview or focus group.
- Robust thematic analysis produced in-depth insights into young people’s agency in improving their peer community’s mental health and well-being.

## Background

 Mental health challenges are the main cause of health-related disability faced by young people worldwide^1^ and constitute a significant burden in low- and middle-income countries.^2 3^ Brazil, a country of continental proportions, is particularly affected. Among 13- to 17-year-olds, who make up over a tenth of the population, one in three experience common mental health challenges.^4^ Difficulties are especially prevalent among high-school aged adolescents^4 5^ and deep-seated inequities in healthcare exacerbate these challenges.^6 7^ Mental health stigma also remains a significant concern.^8^

Unfortunately, the mental health needs of young people in Brazil are largely unmet, with only a small percentage of adolescents in Brazil receiving appropriate care as services often fall short.^9^ Neglecting young people’s mental health results in enduring impacts—for example, around three-quarters of lifelong mental health issues begin by the age of 24.^10^ Although Brazil has recently seen important policy reforms aimed at improving mental healthcare, the focus remains predominantly on treatment.^11 12^ Crucially, effective solutions should also be preventative, and centre on the promotion of good health and collective well-being.^13^

A promising strategy for developing solutions is integrating young people as partners in promoting their own mental health, and that of their peer communities.^14^,^17^ Such a participatory approach aligns with a broader shift in the way researchers, practitioners and policymakers involve young people in their work. Rather than ‘the problem’, young people are increasingly being recognised as part of the solution.^18^ This shift was prompted in recent decades by legislations and treaties around the world that frame young people as rights-bearing subjects, active players in social life and as having a transformative potential to promote societal change and social justice.^19^,^22^

### Youth participation in mental health promotion

The process by which young people partake in defining, understanding and solving issues concerning their lives, communities and society at large, is known as ‘youth participation’ or ‘youth protagonism’.^23 24^ Youth participation is a means of social justice promotion which involves the democratic participation of young people according to their individual and shared needs and interests, both within and outside their immediate social contexts and broader communities.^25 26^

A recent survey suggested that adolescents in Brazil identify schools and community spaces as their preferred settings to positively impact mental health.^14^ Indeed, these spaces constitute a significant part of young people’s lives and therefore have great potential for providing opportunities for youth protagonism and mental health actions.^27^,^29^ In terms of supportive relationships, young people tend to trust their peers and rely on them for emotional support and information. Peer-to-peer interactions can have a significant impact on communities of young people, such as by reducing stigma and encouraging health equity.^16 30 31^ Given many young people’s creativity and unique understanding of their peers’ needs and priorities, they may be in a position to offer acceptable and effective solutions.^32 33^ However, it is yet unclear to what extent young people feel a sense agency and responsibility towards promoting their peers’ mental health and well-being.

### Agency, responsibility and an ecological approach

‘Agency’ and ‘moral responsibility’ are broad concepts with philosophical complexities that are beyond the scope of this study. Here, we define these concepts in the context of our particular research aims and interests. We understand ‘agency’ to be the ability to make purposeful choices and to shape future possibilities.^34 35^ We view ‘moral responsibility’ as the commitment to act in ways that contribute positively to the welfare of others and the community, such that involvement in actions consistent with these concerns reflect and influence one’s moral character.^36^ A sense of agency and moral responsibility can help one to become an active player in civil society—not merely as a recipient of external resources, but as a source of motivation towards social transformation and social justice.^36 37^ However, taking on these roles relies on supportive community structures to ensure that young people’s engagement is safe and sustainable.

According to an ecological framework of participation,^38^ young people’s ability to support their peer communities’ mental health and well-being is influenced by a web of interrelated factors, both internal and external. In other words, agency and participation do not occur in a vacuum; different conditions and environments offer different opportunities for young people to exercise their agency. Indeed, existing literature on civic engagement suggests that young people face several barriers that limit their ability to engage in civic action, including: societal prejudice towards young people, asymmetrical communication dynamics between youth and adults, lack of resources (e.g., time, transportation), insufficient knowledge or information regarding elements pivotal to civic engagement, social-emotional factors (e.g., not knowing how to approach others or being afraid of how they will respond) and limited opportunities to engage.^39 40^

Despite this growing literature, little is known about the specific factors that help or hinder Brazilian young people to engage in actions promoting mental health among their peer communities. It is critical to understand their own views on these factors to support the development of adequate and safe approaches to youth participation in supporting community mental health. This is especially important in Brazil, a country with high cultural and ethnic diversity that varies significantly across different communities. Moreover, the recent dismantling of the children’s and adolescents’ rights agenda in the country has exposed young people to serious violations and restrictions.^41 42^ Given concerns that existing literature on youth participation may not fully reflect the realities in the Global South,^43 44^ empirical research is imperative. Gaining a deeper understanding of the lived experiences of young people in these regions can illuminate their perspectives and lead to their meaningful participation in efforts towards improving mental health in Brazil.

## Goals

This study aims to investigate Brazilian young people’s perspectives on increasing or improving their peer community’s mental health and well-being. We investigated the following questions: (1) How do adolescents perceive their sense of agency and responsibility in promoting their peers’ mental health and well-being? and (2) How do young people perceive the conditions and contexts affecting their prospects of participation in promoting their peers’ mental health and well-being? In order to gain an in-depth understanding of adolescents’ perspectives we used a qualitative methodology co-produced with adolescents, employing Braun and Clarke’s^45^ reflexive thematic analysis (RTA) to explore patterns of meaning through the lens of our collective knowledge and experiences.

## Method

### Patient and public involvement

This study applied a co-production model, following key principles of inclusivity, power-sharing and reciprocity.^46^ Senior researchers and youth collaborators worked together to plan and deliver the study across the whole research cycle. The Youth Collaborative Group (YCG) consisted of five Brazilian young people aged 17–21 years who shared an interest in community engagement and mental health, all of whom coauthor this manuscript. The YCG codesigned the focus group/interview guide, designed materials for recruitment, led the focus groups/interviews, participated in the interpretation of findings and edited this manuscript. Further information and discussion regarding our co-production approach is presented in Siston *et al*.^47^

### Participants

This study had 46 participants aged between 15 and 18 years old—the mean age was 16.4 years old (SD=0.90). 35 of them identified themselves as ‘woman/girl’ (32 cisgender and 3 trans), 6 as ‘man/boy’ (all cisgender), 1 participant declared themselves as non-binary, another as gender-fluid and 3 preferred not to say. Regarding sexual orientation, 21 participants declared themselves as straight, 18 as bisexual, 4 as gay/lesbian and 3 as pansexual. Brazil has five geopolitical regions and participants were represented as follows: 12 from the South, 11 from the Southeast, 9 from the Northeast, 8 from Centre-west and 6 from the North region. 29 participants self-identified as black or having a mixed-race background, and 7 as white, using the Brazilian census criteria. 38 studied in state schools and 8 in private schools.

### Materials, procedures and data collection

Recruitment of participants was conducted primarily via adverts and posts on Instagram aimed at young people resident in Brazil. We used snowball sampling and liaised with schools and organisations (we asked them to share study information with an invitation to participate). A sampling matrix was used to ensure representation from the five regions of Brazil and a minimum of two men and two women from each region. The data collection process considered the data’s *information power*, that is, ‘the more relevant information a sample holds, the fewer participants are needed’ (p210).^48^ We believe the matrix, along with the richness of the collected data, enhanced the representativeness and relevance of the dataset.

Young people were directed to an electronic form in which they could express their interest in taking part in the study. The team then contacted potential participants to provide further information regarding the study as well as information regarding parental consent and young people’s assent. Our recruitment success rate was one participant recruited among five who expressed interest (considering the number of no replies, no shows, consent not given by parents or assent not given by young people).

**Figure 1:**
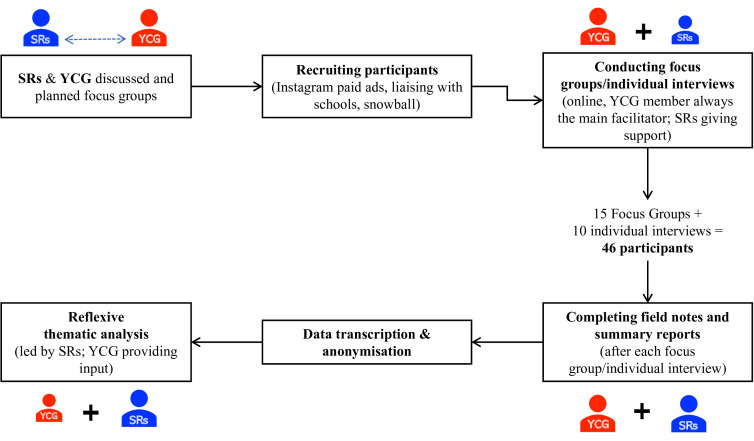
Collaborative data collection process. SRs, Senior Researchers; YCG, Youth Collaborative Group.

Data collection took place between November and December 2021. It was carried out online due to the COVID-19 pandemic but also to allow participants from across Brazil to take part in the study. As seen in figure 1, 15 online focus groups and 10 online individual interviews were conducted, all in Portuguese. We conducted focus groups as well as interviews to retain participants when only one participant attended the day/time scheduled for the focus group. Although not previously planned, the combination of focus groups and individual interviews allowed us to collect comprehensive data on participants’ personal experiences, perspectives and beliefs, which enhanced our data richness and, subsequently, the results’ trustworthiness.^49^

The whole research team, consisting of senior researchers and the YCG, co-produced materials for the study. The main facilitator during the focus group and individual interviews was always a researcher from the YCG, with senior researchers taking a supportive role. Each session started with a brief icebreaker, followed by general questions about mental health and motivation for participation (eg, ‘What or who needs to change for young Brazilians to have good mental health?’; ‘Would you like to contribute to these solutions?’). Participants were then asked to react to a vignette—produced by the YCG—where a schoolmate (named ‘Noah’) shows early signs of mental health challenges. Questions revolved around responsibility (‘As a young person, would you feel responsible for supporting Noah’s mental health?’) and ecological influences (‘Do you feel that you would be able to help Noah?’; ‘Is there anything that motivates/demotivates you to help other young people?’). A translation of the full focus group/interview guide is provided in online supplemental material 1 and the vignette is available online https://www.youtube.com/watch?v=5nk9i0P0Iw4), with an English translation provided online https://www.youtube.com/watch?v=y3be165iPYE).

During and after each focus group/individual interview, facilitators completed field notes and provided a summary report highlighting the key discussions and insights (see online supplemental material 2 for protocol). Interviews were audio recorded and transcribed in full; after transcription, all documents were anonymised. Focus groups had an average length of time of 111 min, varying between 66 and 135 min, and the average amount of words per transcript was 11 704 (ranging from 6189 to 16 742 words). Interviews had an average length of time of 63 min, varying between 33 and 110 min and the average amount of words per transcript was 7428 (ranging from 4321 to 13 669 words).

### Data analysis

We employed an RTA, a method for identifying, analysing and reporting patterns (themes) within data, which emphasises researchers’ valuable subjective role in the processes of data collection and analysis.^45 50^ This approach aligned well with the co-productive, reflexive nature of our project, which entailed ongoing discussions, critical inquiry and open dialogue concerning our interactions and positionality throughout the project. The analysis acknowledged researcher subjectivity and critical reflexivity, as well as the surrounding cultural context.^51^ The data analysis was conducted using the original Portuguese-language data. Therefore, only the excerpts presented in this paper have been translated.

All authors involved in data analysis and interpretation assessed their roles, perspectives, experiences and motivations regarding our research project and its context, as well as the topics of mental health and youth protagonism, in a reflexivity exercise.^45 50 52^ This included a collective reflection during weekly meetings followed by an individual writing exercise, a summary of which is available in online supplemental material 3.

The process of data analysis was inductive, and comprised six phases, inspired by Braun and Clarke,^45^ and Nowell *et al*,^53^ as adapted by de Alcântara Mendes and Ormerod^51^:

- *Phase 1: Familiarisation*—all authors prepared preliminary reports including rich field notes (containing verbatim quotations and paraphrases) as well as impressions and insights after conducting each focus group or individual interview. Before starting to code, the first author went through these preliminary reports and field notes so they could be ‘familiar’ with the data and its patterned meanings.
- *Phase 2: First level of analysis and open coding*—the first author organised, described and categorised full transcripts in an ‘open way’ based on the research questions, using NVivo V.1.6.1 (see online supplemental material 4 for the nine initial codes generated).
- *Phase 3: Second level of analysis and generating initial themes*—the first author generated four ‘candidate themes’ and eight features (or ‘subthemes’). Through discussion, the team helped to improve coherence as well as relevance to the research questions (online supplemental material 5).
- *Phase 4: Reviewing and setting themes*—candidate themes and features were further improved, generating four final themes and 11 features.
- *Phase 5: Anchoring themes and thematic map*—each theme was anchored to their units of analysis (ie, participants that contributed to generating that theme). In this phase, we also developed a thematic map showing how themes were connected.
- *Phase 6: Trustworthiness*—to further enhance credibility and dependability,^54^ we conducted ‘member reflections’.^50 52^ Participants were recontacted via email or text and sent an open-ended survey. The survey began with a video summary of the results produced by the YCG. Following the video, participants were asked questions to reflect on the relevance and significance of the results, to consider whether the results accurately represented their views and to provide their own interpretations of the findings (see online supplemental material 6 for protocol).

Throughout phases 1, 2 and 3, the first author used ‘memoing’ to take notes of ideas, insights and interpretations generated during the analysis process. This tool was important for identifying connections and patterns of meaning across the dataset.

The study’s ‘unit of coding’ (segment of raw data considered as a basic unit of the data analysis) was sentence-by-sentence and the ‘unit of analysis’ was each participant. There are three approaches to analysing data from focus groups^55 56^: individualised data (ie, considering each participant), group as a whole (ie, the whole groups is considered as a unit of analysis) and group interactions (ie, the analysis focuses on the interaction among participants, highlighting their agreements and disagreements on the topic discussed). However, there is no consensus on which approach is most appropriate.^56^ Considering our research questions (which focused on the young people’s personal attitudes and experiences regarding promoting others’ mental health), we decided to consider the ‘individualised data’ as our unit of analysis. Despite this approach not focussing on degrees of consensus and dissent, we believe our (sub)themes reflect the various distinct views of the young people about promoting community/peer mental health.

## Results

**Figure 2:**
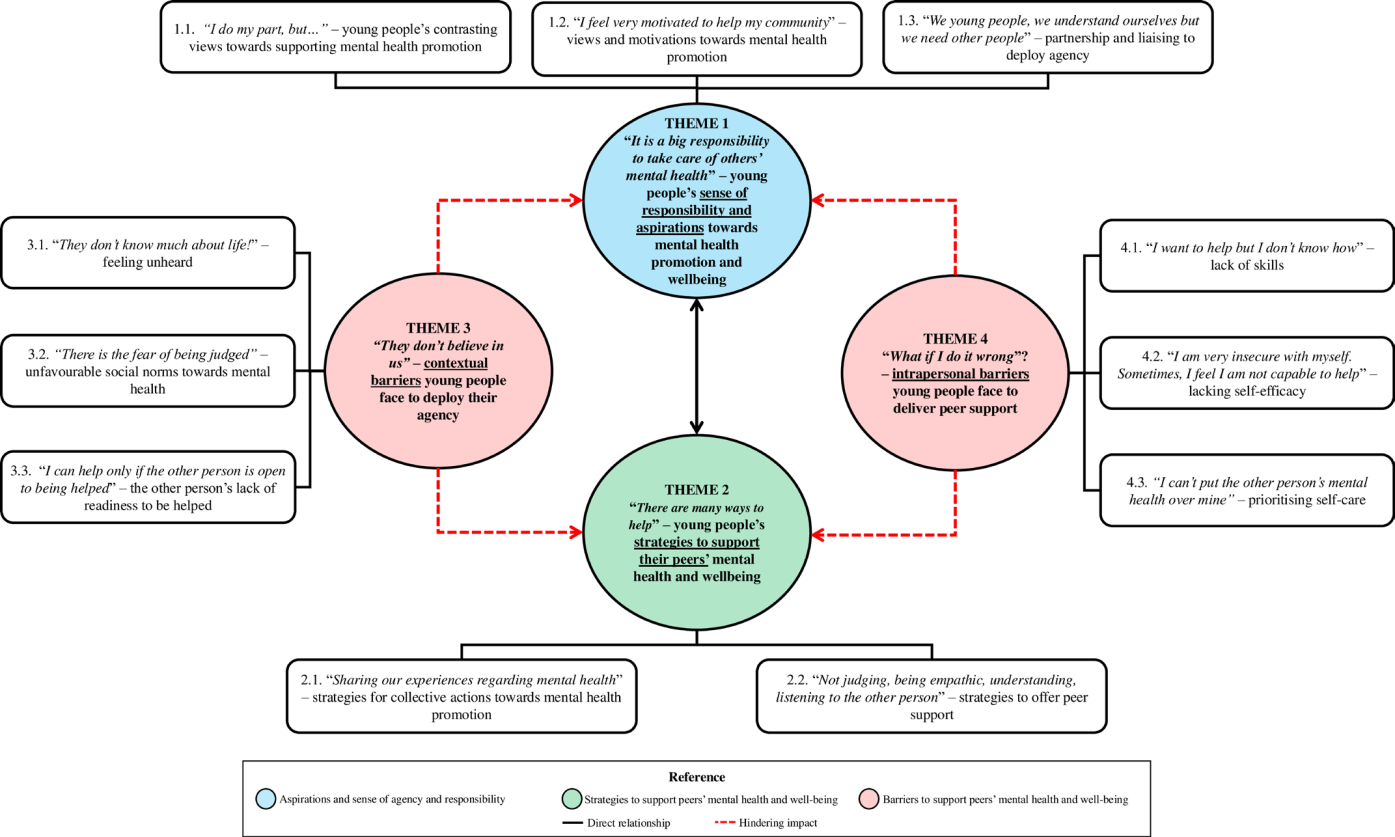
Thematic map of data.

A thematic map is presented in figure 2, providing an overview of the themes and their features. The core themes revolved around young people’s sense of responsibility and their aspirations to support their peers’ mental health and well-being; the strategies they currently employ or wish to use for this purpose; and barriers, encompassing both contextual and intrapersonal factors, preventing young people from promoting mental health and well-being within their communities. Table 1 presents how the themes and their features are anchored in the data. Each participant is represented by an ID formed by the letter ‘P’ followed by a number, for example, P1. Rather than a quantitative measure of significance, the anchoring strategy shows a trace of the dialogic process between participants and the researchers’ subjectivities and provides transparency (ie, trustworthiness).^45 50^

**Table 1:** Themes and features generated by the reflexive thematic analysis and their anchoring on the data

Theme	Anchoring
**Theme 1: ‘It is a big responsibility to take care of others’ mental health’—young people’s *sense of responsibility and aspirations* for mental health promotion and well-being**	
Feature 1.1: ‘I do my part, but …’—young people’s contrasting views towards supporting mental health promotion	P1, P2, P4, P5, P6, P9, P10, P11, P12, P13, P14, P15, P16, P18, P19, P20, P21, P24, P25, P26, P27, P31, P33, P34, P35, P37, P42, P43, P44, P45, P46
Feature 1.2: ‘I feel very motivated to help my community’—views and motivations towards mental health promotion	P1, P2, P3, P4, P5, P6, P7, P8, P9, P10, P11, P13, P16, P17, P18, P19, P24, P25, P29, P31, P33, P35, P36, P42, P43, P44
Feature 1.3: ‘We young people, we understand ourselves but we need other people’—partnership and liaising to deploy agency	P1, P2, P5, P11, P13, P18, P28, P32, P33, P34, P45, P46
**Theme 2: ‘There are many ways to help’—young people’s *strategies to support their peers’* mental health and well-being**	
Feature 2.1: ‘Sharing our experiences regarding mental health’—strategies for collective actions towards mental health promotion	P1, P2, P3, P4, P5, P6, P7, P8, P9, P10, P11, P13, P16, P17, P18, P19, P24, P25, P29, P31, P33, P35, P36, P42, P43, P44
Feature 2.2: ‘Not judging, being empathic, understanding, listening to the other person’—strategies to offer peer support	P1, P2, P3, P4, P6, P7, P8, P9, P10, P11, P12, P13, P14, P16, P17, P18, P19, P20, P21, P22, P23, P24, P26, P27, P28, P31, P32, P33, P34, P36, P38, P39, P40, P41, P42, P45, P46
**Theme 3: ‘They don’t believe in us’—*contextual barriers* young people face to deploy their agency**	
Feature 3.1: ‘They don’t know much about life!’—feeling unheard	P1, P6, P,7, P13, P14, P15, P16, P17, P18, P19, P20, P21, P22, P28, P29, P33, P34, P35, P36, P42, P44, P45, P46
Feature 3.2: ‘There is the fear of being judged’—unfavourable social norms towards mental health	P1, P5, P14, P17, P19, P25, P26, P31, P37, P38, P40, P41, P42
Feature 3.3: ‘I can help only if the other person is open to being helped’—the other person’s lack of readiness to be helped	P10, P11, P12, P13, P14, P16, P17, P19, P25
**Theme 4: ‘What if I do it wrong?’—*intrapersonal barriers* young people face to deliver peer support**	
Feature 4.1: ‘I want to help but I don’t know how’—lack of skills	P1, P3, P4, P5, P7, P9, P11, P15, P16, P17, P24, P25, P26, P27, P28, P30, P31, P44, P45, P46
Feature 4.2: ‘I am very insecure with myself. Sometimes, I feel I am not capable to help’—lacking self-efficacy	P2, P9, P13, P16, P17, P18, P20, P21, P22, P24, P25, P26, P27, P33
Feature 4.3: ‘I can’t put the other person’s mental health over mine’—prioritising self-care	P1, P2, P9, P10, P11, P12, P13, P14, P16, P17, P19, P24, P25, P26, P27, P28, P33, P34, P35, P41, P42, P43, P44, P45, P46

We acknowledge Braun and Clarke’s critiques^50^ regarding thematic conceptions and titles that seem to be more a topic summary than ‘interpretative stories built around [a] uniting meaning’ (p3). However, we also acknowledge the need to make results derived from qualitative inquiries helpful for policymakers and decision-makers more broadly, who may be more likely to engage with pragmatic, descriptive outputs,^51 52^ the reason for which our thematic elements have descriptive titles. As a remedy, we prioritised ‘in vivo coding’ for themes’ and features’ labels, with an exploration of deeper meanings within features. A youth-friendly video summarising the results can be accessed online (https://www.youtube.com/watch?v=P1Cmk3SERjA (Portuguese version) or https://youtu.be/6827368TsOM (English version)).

### Theme 1: ‘It is a big responsibility to take care of others’ mental health’—young people’s sense of responsibility and aspirations towards mental health promotion and well-being

Young people acknowledged that supporting their peers’ mental health and well-being is a complex task and expressed divergent perspectives regarding their sense of responsibility in this area, as shown by feature 1.1. Notably, some participants expressed that supporting their peers’ mental health was not their responsibility:

I do not think it is my responsibility [to support young people’s mental health] because it is a big responsibility to take care of someone’s mental health. Therefore, I just feel responsible for my own mental health, which is already a lot. (P33)

Apart from the burden of looking after each other’s mental health, another participant thought the government had an important duty to take responsibility for public mental healthcare: ‘I will do my part as a human being but I believe it [supporting young people’s mental health] is a responsibility of the State, the society and it is everybody’s duty in the Brazilian Constitution’ (P26). These quotes suggest a view of participation in promoting mental health as a collective form of action—not a product of an individual’s effort but rather a collective one. They also allude to the risk that supporting youth mental health can be felt as a burden for some young people, and is a responsibility that should not be placed (solely) on them.

Nonetheless, many young people did express a sense of responsibility and commitment to providing peer mental health support: ‘I always feel responsible [for supporting other young people’s mental health]’ (P15); especially for friends: ‘I feel very responsible for my friends (…) if I can do something to help, I will’ (P5). These excerpts highlight the role of friendship-based obligations in driving young people’s sense of responsibility in the promotion of mental health. In this context, participation emerges as a result of caring inter-relational processes aimed at nurturing valued social bonds.

Despite their varying sense of responsibility in relation to promoting their peers’ mental health, most participants demonstrated motivation to engage in actions to promote the mental health and well-being of their peers and community, as evident in feature 1.2. Some participants emphasised their unwavering motivation, often driven by their personal characteristics and experiences. ‘I feel very motivated to help my community not only regarding mental health issues but also regarding other areas’ (P24). Another shared, ‘I am a more introverted person but whenever I see someone struggling, no matter if they are my friend or not, I go there and try to help that person by doing whatever I can’ (P1). Additionally, another participant noted, ‘I have been through some struggles and I did not have support from my school, community, or health services. This is why, whenever I can help someone going through mental health issues, I do help them’ (P4). These excerpts underscore the inter-relational nature of engaging in mental health actions, which aligns with the focus of feature 1.1. They reveal a commitment to community involvement and fostering positive interactions, highlighting youth participation as an ongoing and reciprocal process rooted in social interactions.

For young people in this study, recognising responsibility and agency and being motivated were not necessarily enough to engage in actions towards the promotion of mental health and well-being. As feature 1.3 shows, young people expressed a need to liaise with others and to establish partnerships to deploy their agency towards supporting their peers’ mental health and well-being: ‘We young people, we understand ourselves but we need other people … there has to be [more people involved] to move forward [the mental health promotion]’ (P1). In this sense, the role of educators seems to be pivotal: ‘A teacher used to support our project but, when she left the school, we discontinued the project because she was the only person who believed in and supported us’ (P33). These quotes reveal that young people’s agency and transformative potential must be ecologically supported by partnering with other young people and with adults, such as teachers.

To conclude findings from theme 1, young people’s sense of responsibility to participate in peer support and mental health actions, as well as their proactive motivation, are prompted and sustained by the (ecological) inter-relations young people maintain with their peers, adults and community contexts.

### Theme 2: ‘There are many ways to help’—young people’s strategies to support peers’ mental health and well-being

Theme 2 captures the multiple and varied strategies employed by young people to support the mental health and well-being of their peers at school and in their community. Some young people referred to one-on-one actions, and others highlighted actions aimed at collective well-being. Feature 2.1 captures their various strategies to enhance *collective* well-being. These actions included reflexive conversations and sharing with others (‘Sharing our experiences regarding mental health with other young people at school like a conversation circle’ (P6)); artistic expressions (‘I am a poetess so I write some poetry and post it on my Instagram. A lot of people DM me saying my poetry made them feel good and this comforts me’ (P3)); positive social media engagement and referrals (‘I would post on social media content regarding places where young people could find help. For instance, in my city, there is an Olympic centre in which people can play sports’ (P7)); and access the communities’ sources (‘At my church, we have a space in which we can open up about ourselves and our problems’ (P35)).

As illustrated above, there was versatility in the way young people felt they could support their peers’ mental health and well-being. Young people were able to harness skills related to listening and sharing in an empathic way, as well as to recognise and signpost sources of help, using a range of creative strategies. Feature 2.2 indicates that similar strategies were suggested by young people regarding direct, one-to-one peer support. For instance, young people mentioned being empathic and open to listening as well as validating the other person’s feelings as forms of emotional support: ‘Not judging, being empathic, understanding, listening to the other person. Sometimes, a simple chit-chat can mean a lot to that person’ (P1). Other young people mentioned social support strategies related to leisure, relaxing and unwinding: ‘There are other ways to help, for instance: sharing a song, going out to play football, eating out, trying to have some leisure and having fun somehow’ (P2).

Participants also mentioned more practical strategies related to helping their peers with school assignments (‘I could help him to finish his assignments if I see he is swamped with readings and assignments. It would not hurt if I help him to complete three assignments’ (P27)), and to amplifying the other person’s social network and referring them to professional help (‘I would try to introduce my friends to Noah [the character presented in the vignette], people he could talk with. Also, I would try to do some activities with him and encourage him to see a psychologist’ (P16)). These strategies illustrate young people’s diverse approaches when it comes to providing one-on-one support.

### Theme 3: ‘They don’t believe in us’—contextual barriers young people face to deploy their agency

Young people in this study referred to several contextual factors that could impair their transformative potential or that could impede or make it difficult for young people to deploy their agency to support mental health promotion, captured here in theme 3.

Feature 3.1 showcases two key inter-relational and contextual barriers that can hinder young people’s agency in supporting the promotion of mental health and well-being: (1) not feeling heard by adults: ‘Adults say like ‘*young people don’t know much about life*’. That is bad because young people have a lot to say, to contribute, but there is not much opportunity for that [for youth participation]’ (P1) and (2) not feeling understood or validated by others after being ‘heard’, especially by adults: ‘When people do not acknowledge what I think, this is a barrier. Some people cannot understand me, what I want to express and this comes not only from my family, it comes from everybody’ (P20); ‘I felt I was talking, talking and nobody was listening to me’ (P22).

According to some of the participants, adults tended to disregard not only young people’s lived experiences but also their capabilities to promote transformative changes. For instance, some mentioned a prevalent attitude among adults to promptly dismiss or distrust young people: ‘There is an ‘army of adults’ doubting our capabilities [to use our agency]’. Some participants described that young people are frequently invalidated or disenfranchised by adults, who often say: ‘he is not capable of doing that’ (P15). This invalidation undermines young people’s basic abilities and rights, as one participant expressed, ‘Just because we are young people, they [adults] think we are not capable of speaking about our own rights, we are not capable of standing for our own mental health’ (P14).

Alongside the systematic invalidation, feature 3.2 shows that young people also faced an unfavourable context that disregarded mental health issues or instilled a fear of judgement, leading young people to falter: ‘Young people judge one another very frequently’ (P5); ‘There is the fear of being judged as mental health is still seen as taboo by some people. Therefore, I found it very difficult to talk about it [mental health]’ (P38). Some young people even feared they could be targeted by bullies if they decided to provide support for someone going through mental health struggles: ‘I also used to struggle to get along with others, and I was bullied too. So, I did not do anything. I did not help him (…) I was afraid of being targeted too’ (P25). The prejudice and discrimination related to sexual orientation and gender identity were also mentioned as prompting an unfavourable environment for young people’s participation: ‘When the people from the council city found out the project was run by transsexual young people, they started to ignore us, they stopped replying’ (P42).

As highlighted by feature 3.3, another common contextual barrier was when the other person is not open or ready to receive support. As one participant pointed out, ‘one can help only if the other person is open to being helped. Some people do not seek help or they are too shy, too introverted to talk about their issues … in these cases, it is difficult to help’ (P10). Another shared that, ‘sometimes, one wants to help but the other person does not want to be helped, even though they know they need help’ (P19).

Taken together, these statements reflect multiple interpersonal and contextual barriers that can affect young people’s agency towards mental health promotion and peer support.

### Theme 4: ‘What if I do it wrong?’—intrapersonal barriers young people face to deliver peer support

This theme captures intrapersonal issues that can impede or make it difficult for young people to take action to support their peers’ mental health and well-being. According to feature 4.1, one such intrapersonal barrier was the lack of supporting skills. This included not knowing about the specific problem or its severity; as one participant shared, ‘sometimes, one does not help because they don’t really know what is going on. Sometimes, they don’t help because they don’t see the situation as very serious or attention-worthy’ (P4). Others alluded to a lack of knowledge of what kind of help one should provide: ‘Sometimes, the problem is not knowing how to help. Many young people are willing to listen to others but they don’t know what to do. Sometimes they withdraw because they do not know how to help’ (P3). Finally, some young people expressed concerns about being invasive or disturbing their peers. As noted by a participant, ‘I want to help but, at the same time, I don’t want to be invasive, to disturb the other person … I don’t want to reach out to the person to offer help, I want them to reach out to me’ (P5).

In tandem with a perceived lack of skills, some young people also did not see themselves as emotionally capable of offering appropriate support to their peers, as shown by feature 4.2. For instance, some of them were worried their intervention could make the situation worse:

I was freaking out and afraid: Oh my God, what if I say something wrong and she kills herself? I was thinking: what if I am pressuring her too much? What if I am asking too much? What if this is not the right advice? (P30)

Some of them also pointed out the anxiety related to peer support: ‘I was anxious … I was afraid to say something wrong and make things worse’ (P18). Some young people just did not feel confident enough:

I do have the will to help others but it is a very difficult task. It is very hard and requires a lot from us, young people. So the question is: am I prepared to do this? Until when can I help? (P13)

The lack of skills and self-efficacy seemed to emerge as critical barriers to young people’s involvement in supporting their peer’s mental health. Furthermore, young people talked about the importance of regulating their efforts to prevent potential negative impacts on their own mental health or the risk of creating dependency among those who receive support, as exemplified in feature 4.3: ‘I can help within my limits, I can do what my mental health allows me to (…) I cannot prioritise the other person’s mental health over mine’ (P14). Young people demonstrated awareness of their own limits to support others’ mental health and some signalled the importance of refraining from offering help when they themselves feel overwhelmed by the situation. For instance, one participant shared that, ‘my limit is when I start to feel overwhelmed by the other person’s issues. Whenever I feel like that, I know I cannot help that person anymore’ (P44). Others referred to instances where the other person started becoming dependent on that support:

When you get too involved, there is a problem because it becomes too heavy and difficult for you and for the other person you are trying to help. This is why, [before helping others] one has to take care of their own mental health. (P34)

A good cue to read the situation was, as pointed out by one of the participants, when the other person started oversharing and expressing irritation whenever they were not there for them:

[The limit for offering peer support is] when the person starts to share things I should not know and then they start to depend on me to feel well. So if they do not talk to me, they feel irritated and feel bad (…) this is the time when I have to tell the person to seek professional help. (P25)

In sum, this theme illustrates the complex intrapersonal factors—including barriers of self-doubt, lack of skills and fear of causing harm, but also awareness of and respect for one’s own needs—that underlie young people’s choices on whether to offer support.

### Member reflections

During member reflections, the vast majority perceived the themes and features identified as highly relevant to their experiences and reported that they felt represented. Many emphasised the external barriers to youth participation; for instance, one participant reflected that ‘the most difficult barrier is the fact that some people still think that mental health problems are bullshit […] or that these issues don’t need to be addressed or solved urgently’. A few participants referred to the fact that ‘young people help each other in any way they can’ as a particularly meaningful result. They also offered reflections on the applied value of the results, and the importance of research that listens to young people’s voices: ‘[The results were] super relevant, showing people how young people feel about their health is extremely important for those outside the bubble of the new generation, after all, they need to understand that the new generation has an opinion and wants and needs to be heard’. For a compilation of participants’ reflections, see online online supplemental material 7.

## Discussion

In this co-produced qualitative study, Brazilian adolescents consistently expressed motivation to support their friends and community through various means. The main strategies mentioned were direct peer support (eg, comforting, signposting and leisure activities) and initiatives to enhance collective well-being (eg, organising discussion circles, arts-based initiatives). While some participants did not feel a sense of responsibility towards their peers’ mental health, the majority did, particularly within friendships. Critically, young people emphasised the formal roles and responsibilities of institutions like schools and government bodies in supporting youth mental health. They also acknowledged that helping others is a complex task that can come with emotional challenges. Participants outlined multiple barriers to deploying their agency towards the promotion of mental health and well-being. Intrapersonal issues were mentioned, most notably lack of peer support skills and confidence to support others. Among external factors, the most commonly mentioned were mental health stigma, adultism and peers’ unwillingness to receive help. Finally, young people emphasised the importance of partnership and support from adults in overcoming these barriers and meaningfully engaging in promoting community mental health.

These findings align with an ecological model of youth participation.^38^ This perspective views youth agency as not a stable trait young people ‘possess’, but rather as a dynamic process influenced by factors within and around them.^57^ This holistic approach was pervasive in the way young people perceived their sense of agency in promoting mental health and well-being, perceived as being influenced by both immediate and more distal resources they might have or lack access to. From a practical standpoint, this suggests that, in order to deploy their agency to promote mental health, young people need training and support. Many mental health interventions involve connecting young people with others of similar age and/or experiences for support-giving; yet, without appropriate training from adults, the support they provide might be ineffective (see^58 59^ for reviews) or even potentially harmful to both parties. Young people in our study highlighted the importance of skills such as being able to properly read another person’s signs, knowing the right time to offer assistance without being invasive and knowing how to best support them. Training these skills can help young people feel more confident in the support they provide and in their ability to support their communities more broadly.^15 60^

In addition to capacity-building, our data suggest that young people’s engagement in mental health promotion should be ecologically supported by a favourable context as well as by fostering partnerships and collaborations with adults. These results are consistent with a previous UK-based study that found adolescents often rely on teachers for mental health education^61^ as well as a global survey suggesting that young people wish to be supported and trained in order to make a difference in mental health.^14^ It is important, however, to strike the right balance between youth and adult involvement. There has been some discussion around optimal models of youth engagement across varying degrees of youth and adult engagement.^18 62^ Adolescents in our study seemed to favour a pluralistic model, whereby adults and adolescents share control.^18^ Pluralistic models have been shown to be successful, for instance, The New Mentality programme in Canada^63^ and the Uplift programme in the UK^16^ established partnerships between young people and adults to promote mental health awareness and well-being, leading to a range of positive benefits for young people.

A partnership approach offers greater protection for young people, given some of our participants’ portrayals of participation in promoting mental health as a ‘heavy duty’ and reports of lack of confidence in their skills or handling the emotional burden. The emphasis on the role of adults as collaborators and protagonists is consistent with Gal’s^38^ and Sutterlüty and Tisdall’s^57^ understanding of youth participation as a relational construct, whereby participation is constrained or motivated by the young person’s interactions with adults and their respective roles and positions. This model seems to be sensitive to the Global South, and particularly Brazilian culture’s focus on interdependence, family relationships and intergenerational dialogue.^43^ Within this partnership model, it is crucial that adults tend to the ways in which young people themselves wish to deploy their agency (including respecting the desire *not* to participate^64^). The diversity of strategies mentioned by young people, from social media engagement to arts-based approaches, signpost the value of youth–adult partnerships that seriously consider young people’s priorities.

Partnership requires openness and trust from adult stakeholders^65^; nevertheless, a common theme in our study was adults’ negative beliefs, attitudes and behaviours towards youth, which left them feeling unheard or invalidated. Adultism is a pervasive problem worldwide^66^ and, unless carefully addressed, can severely hinder young people’s civic participation,^39 40^ which is a loss for all generations of society. This underscores the importance of providing not only young people but also adult stakeholders with reflexive resources and tools to help overcome barriers to youth participation. Additionally, urgent attention is required for implementing larger policies in schools and nationally that promote youth participation, while addressing historical and social constraints such as identity marginalisation.^67 68^

Similarly, unfavourable social norms towards mental health are common among adults and young people across the world,^69^ including Brazil.^70^ As our data suggest, mental health stigma might lead young people to withdraw from engaging with the topic or supporting their peers’ mental health (even if motivated to) because they fear being judged by others, or even targeted by bullies. Policies that promote mental health awareness can contribute to a more supportive environment for young people’s agency in the Brazilian context. These policies must also consider the intersectionality of mental health stigma with other forms of oppression that young people encounter, including racism and anti-LGBTQ+ bias,^69^ which were part of the experiences of young people reported in the current study.

Critically, in addition to one-to-one strategies such as emotional support, young people also aspired to promote collective well-being, for instance by acting on social determinants of mental health and promoting a positive school climate. Indeed, empowering young people to participate effectively involves not only building their skills but also developing their critical thinking about sociopolitical conditions, enabling them to challenge oppressive social structures and drive social change.^71 72^ Encouraging participation in health promotion without considering these social structures can in fact disempower disadvantaged groups and potentially worsen health inequalities.^73^

### Strengths, limitations and future directions

Our study was fully co-produced with young people. Through a strong youth-researcher partnership, we developed engaging and accessible research materials to conduct interviews and focus groups that enabled adolescents to provide rich responses to the project. These data were systematically analysed by experienced qualitative researchers, drawing on an ecological framework of youth participation, with input from youth coresearchers and research participants. Through online recruitment, we were able to engage adolescents across Brazil’s five macro-regions. Participants were mostly women, students from state schools and living in urban areas across the different Brazilian regions, with strong LGBTQ+ representation. Future studies should investigate the perspectives of other groups that were underrepresented in our study, including men/boys, young people from rural areas and those with an Indigenous background, and analyse results from an intersectional lens. A key limitation is that our study sample was likely biased towards those with an interest in mental health and participation, especially since no reimbursement was provided. Conducting future studies in educational settings, for instance, engaging whole school classrooms, can help circumvent this limitation.

Given adult stakeholders’ important role in sustaining youth participation, future research should triangulate the views of parents, teachers, mental health professionals and policymakers. Moreover, the results of this study must be considered with reference to the historical context in which the data were collected. Data collection took place during a health emergency and amid a period of weakening of social protection networks.^42 74^ In this challenging scenario where key stakeholders such as politicians and policymakers fail to meaningfully uphold citizens’ rights and social justice, youth participation in mental health initiatives could feel like a burden to adolescents. Future research should prioritise investigating policies and strategies that ensure adults fulfil their responsibilities to support young people in safely exercising their rights to participation in challenging political and social contexts.

## Conclusions

Through a co-produced study, we found that adolescents in Brazil were motivated to promote their peers’ mental health, not only through direct peer support but also through collective action. However, as strong as young people’s transformative potential may be, their motivation to promote mental health does not necessarily lead to action. The key contribution of our study is highlighting the importance of a favourable context in which they can grow their skills and actively participate. Creating this favourable context starts with challenging adultism as well as social norms that reinforce stigma around mental health issues. It also encompasses partnering with young people and supporting them to develop the personal skills they feel they are lacking to be able to contribute effectively. Within our project, these results helped us to design a digital tool (ie, chat-story) aimed at enhancing Brazilian young people’s skills to promote their peers’ mental health and well-being, which has been disseminated to adolescents across Brazil.^75^

From the emphasis on adult–youth partnerships to the development of a non-stigmatised environment, the main implication of our findings is that youth participation towards mental health promotion must be a collective effort. Adults and organisations interested in fostering youth participation should adopt an ecological perspective. This includes understanding young people’s aspirations as well as the factors that can either facilitate or hinder their engagement in mental health and well-being promotion. Moreover, it is critical to be mindful of the potential risks that young people may face, including emotional demands when providing support to their peers or engaging in activism, and take steps to protect them. Ensuring that adequate services and protections are in place is essential. We hope our findings help pave the way towards mental health initiatives that are centred on empowering young people to safely exercise their right to participation. This approach is not only a way of assuring young people’s best interests are met, but also a robust way to build a fairer and more inclusive society.

## supplementary material

10.1136/bmjopen-2024-084996 online supplemental file 1

## Data Availability

Data are available upon reasonable request.
