# Supplementary material for: Young people’s sense of agency and responsibility towards promoting mental health in Brazil: a reflexive thematic analysis
Source: BMJ Open. 2024 Dec 7;14(12):e084996. doi: 10.1136/bmjopen-2024-084996 (PMC11628997; doi:10.1136/bmjopen-2024-084996)
Supplement: online supplemental file 1 [file bmjopen-14-12-s001.pdf]

Supporting Information for

# Young people's sense of agency and responsibility towards promoting mental health in Brazil: A reflexive thematic analysis

Josimar Antônio de Alcântara Mendes; Sheila Giardini Murta; Felipe Rodrigues Siston; Rafaela de Oliveira da Cunha; Brenda Thallys Rocha Seabra; Julyana Alves Ferreira; Rafa Ribeiro Alves de Souza; Victor Hugo de Lima Santos; Ilina Singh; Gabriela Pavarini*

*Corresponding author: gabriela.pavarini@ethox.ox.ac.uk

**This file includes:**

Supplementary material 1. Focus group/interview guide

Supplementary material 2. Field notes protocol

Supplementary material 3. Reflexivity statement

Supplementary material 4. Initial codes

Supplementary material 5. Second level of analysis

Supplementary material 6. Member reflections

Supplementary material 7. Compilation of participants' reflections

# Supplementary material 1.

Focus group/interview guide

**Supplementary material 1.** Focus group/interview guide

# Focus Group/Interview Guide for Youth Protagonism in Mental Health Promotion: Mapping of Aspirations and Practices

**Expected duration: 2h max**
**Roles:** *Main Facilitator*: Youth Collaborative Group member & *Support Facilitator*: senior researcher
**Support material**: video vignette (Noah)

Wait for a max. of 10 min. to start session

→ Ideas for 'ice breaking': How did you find out about the project? Where are you from? Do you have family or friends in the area?

## Presentation and Icebreaker (15 minutes)

***- Group presentation, name, age, and where they are from***
*Say: our name, where we speak from (city/state) and something unique that only exists in the place where you live (it can be your neighbourhood, your city or state). I'm going to start… my name is…*

***- Facilitators [each pair] explain what a focus group is, and how the process will be***

- ***1st.*** *Respect* ***confidentiality and privacy:*** *everything we say and discuss here should not be shared outside the group. Our discussion will be recorded because our memory sometimes fails (!), but the recording will be confidential. We will then transcribe the conversation and remove any information that could identify you. We will not tell anyone that you participated unless you tell us something that indicates a serious risk to you or others. In that case, we will talk to you and decide together who needs to be informed so that we can protect your health and safety. If at any time you feel uncomfortable, and wish to leave, or have any other problems, just write in the private chat to one of the facilitators.*

- ***2nd:*** *Respect* ***each one's time and space*** *to express themselves freely. We suggest that we use the feature of raising your hand to speak, like this... [demonstrate on video-conferencing platform] and the facilitator will give you the space to talk. When someone is speaking, we ask that we listen to them carefully and with empathy. We can react using emojis or write in chat but please do not interrupt one another verbally. We will share unique stories and perspectives. Don't worry about responding directly to the stories you hear and/or offering advice… This way we can all feel free to express ourselves and*

*our stories, our own thoughts, feelings and ideas, openly and honestly, without judgment or fear*

- *Is there anything else that you think is important for a productive meeting that we did not list?*

**START RECORDING - [SAVE TO COMPUTER]**

**Mental Health Literacy (Visions and Knowing Health Services) [8 minutes]**

1) What's the first thing that comes to your mind when you think of mental health? Do you find it easy or difficult to talk about mental health?

**Motivation and Aspirations [20 minutes]**

2) How do you think young people in Brazil are doing with regards to their mental health? Why?
3) What leads to a good or bad mental health? (if needed) Do you think that elements such as race, gender, sexual orientation, and social class can impact young people's mental health? How?
4) What or who needs to change for young Brazilians to have a positive mental health?
5) Would you like to contribute to these solutions? (If so) In what way?
6) What barriers hinder or discourage your engagement in the community or society as a whole (including projects for better mental health)? (If needed) For example, some young people feel that they don't have opportunities to talk about what they feel, or that they are repressed when they try. Is that you? Any other barriers?
7) How can we overcome the barriers imposed on young people to engage in mental health promotion?

**Thematic trigger [vignette]**

*Now, we're going to discuss mental health a little more, starting from a hypothetical case. We will show you a story on the screen, which should take about 5 minutes. If you can't hear the audio, let me know [share screen with presentation] [**5 minutes**]*

| |
|---|
| **Noah's vignette:** https://youtu.be/y3be165iPYE |

**Engagement and Participation [15 minutes]**

8) What aspects of Noah's story most stand out to you?
9) Have you ever met a young person in a situation of emotional distress like Noah? What were the difficulties he/she experienced?
10) How did you react to the situation? What made you have that reaction? If you haven't had the opportunity to support that person, what stopped you?
11) In this situation, or others that you have experienced, did it occur to you to contact a health service, a teacher, or an adult? How was the experience? Even if you haven't had this experience, would you know who to contact?
12) Do you feel that you would be able to help Noah? If so, what would be your way of helping him? What difficulties could you face along the way? Is there anything that motivates/demotivates you to help other young people?

13) Do you contribute to promoting or supporting the mental health and well-being of young people **in your school?** Could you give **an example**?
14) What about in other contexts like online, in youth groups? What helped you do this? If you did not have the opportunity to provide this support, what were the barriers?

## Agency and Responsibility [15 minutes]

15) Whose duty is it to help Noah? (If needed) For example, parents, teachers etc. Overall, whose duty is it to promote and care for the mental health of young people? (if needed) e.g., teachers, politicians, researchers.
16) As a young person, would you feel responsible for supporting Noah's mental health?
    To what extent **do you, as a young person,** feel it is your duty to support and promote the mental health of other young people in your community?
17) Do you think this support would also be possible through actions within your school or community? In what way?
18) What sources of support or information regarding young people's mental health do you know at your school? And in other contexts?
19) What types of support or information are lacking at school? [What about in other contexts, such as online and in your community?]
20) If change could start within your school, what would be **one way** you would personally like to help? [And in other contexts, such as online or through youth groups?]

## Closing [8 minutes]

*To close our meeting, we would like you to now share the verse of a song, which we requested prior to today's meeting, which inspires you or works as an emotional support, helping you through difficult times. We ask you to read the verse and, if you would like to, you can comment on why you chose this song, in 1 min maximum. I'll start…*

## Debriefing [15 minutes]

We appreciate everyone's presence and participation. Your contributions today will help us build a digital tool, a "chat-story", to support adolescents and help them be "agents of change" and promote better mental health in their schools and communities.

To find out about future, or the chat-story, you can follow our Instagram at @engajada.mente or at www.engajadamente.org.

On our website there is a page where you will find a range of contacts for psychological support in case you need support (or would like to refer someone to support) at any point. We are available for any questions or comments you may have. It was a pleasure to meet you!

**SAVE FILES TO ONEDRIVE + FACILITATORS JOINTLY FIL THE REPORT**

# Supplementary material 2.

Field notes protocol

**Supplementary material 2.** Field notes protocol

# Field Notes Protocol

*Once the focus group is over, please allow 10 to 15 minutes for you both to discuss and fill up the following information and questions. This information/answers will be essential for our Innovation Lab. Therefore, please fill in as completely as possible.*

| FOCUS GROUP/INTERVIEW Nº | |
|---|---|
| Facilitators: | |
| Date: | Number of participants: |
| Time: | City/State: |
| Duration: | |

In general, how do you evaluate the focus group/interview? What about group dynamics?

Did this focus group/interview give you any ideas for the chatbot and the intervention we will co-create?

Free space for comments:

Please, try to summarise participants' accounts for each question from the focus group/interview. Please, use the notes you took during the focus/groups:

## Mental Health Literacy (Visions and Knowing Health Services)

1) What's the first thing that comes to your mind when you think of mental health? Do you find it easy or difficult to talk about mental health?

**Supplementary material 2.** Field notes protocol

## Motivation and Aspirations

2) How do you think young people in Brazil are doing with regards to their mental health? Why?

3) What leads to good or bad mental health?

4) What or who needs to change for young Brazilians to have good mental health?

5) Would you like to contribute to these solutions? If so, in what way?

(...)

# Supplementary material 3.

Reflexivity statement

**Supplementary material 3.** Reflexivity statement

# Reflexivity Instructions

Reflexivity is a process that requires the researcher to reflect on their actions, feelings and perceptions throughout the research process, but especially the data analysis. The aim is to enable recognition of how the phenomena under study affected the researcher, what this process says about the research and how this touches on their history, conceptions and values. The idea is to highlight the subjective role of the researcher and how this took place during the research process and data analysis, i.e. what biases may have entered into the process of investigating and interpreting the data. This is an alternative to a sterile neutrality, which assumes strict orthogonality between the observer (researcher) and object (phenomenon). Reflexivity recognises non-neutrality and its implications for the process of investigating and understanding a research object. If you want to know more, you can read **this paper** or **this one**.

***Instructions to write your 'reflexivity'***:

- Please write between 10 and 15 lines;
- Tell us a little about yourself and how the research topic 'affects' or 'touches' you;
- Talk about how you perceive and understand the main topics of the research (e.g., youth participation; engagement; mental health and youth protagonism, etc.);
- Make it clear what your position is in relation to the participation of young people in social life in general;
- Feel free to write in English or Portuguese

# Reflexivity Statement

**Josimar Antônio de Alcântara Mendes, Senior researcher**

Throughout my career, I have been interested in investigating and comprehending issues related to family life and children's rights and best interests. My interest in children and youth participation has grown in the past few years. I have become an enthusiastic advocate for the recognition of children and young people as subjects of rights with inherent creative and transformative power to promote social justice and societal change. I see children and young people as active members systemically bound to their relational contexts' dynamic, and as contributors to it. I believe the child or adolescent should be seen as a protagonist in social life, rather than as a mere spectator. Moreover, I believe no society can fully develop or function without recognising children/young people as such; without fostering and applying any means possible to ensure children's and young people's complete and protected participation in social life.

**Sheila Giardini Murta, Senior researcher**

My work as a psychologist and researcher in the field of mental health promotion prevention with different populations throughout their lifespan has taught me that mental health is comprised of structural social determinants, not only contextual ones. As a Brazilian, I have witnessed the impact of social inequities on the living conditions of young people, which worsened in recent years due to the adverse political context in the country.  This makes me attribute immense value to youth agency and protagonism for mental health promotion, marked by their engagement in collective actions capable of impacting systems of oppression that affect well-being. School, as the Brazilian educator Paulo Freire would say, is a perfect place to practice hope in action ("esperançar" in Portuguese). Basing myself on this committed hope, expanding opportunities in favour of a better future for youth is, for me, an unavoidable task.

**Felipe Rodrigues Siston, Senior researcher**

In my doctoral thesis in Communication and Culture, I used an autobiographical method to understand the experience of a particular madness. I took the risk of reflecting upon the difference or the mental suffering that accompanied me throughout my youth. However, it was only in my adulthood, when writing the thesis, that I went through a biomedical and self-stigmatizing intervention. Reflecting on the voice and place of a 'Wandering Researcher' in the

production of knowledge and social change led me to cultural strategies of psychosocial care and attention. Amongst them were my participation in the community management of a library recovered by young people, who occupied an abandoned school building, and in the citizen laboratory derived from it; and coordination, within a local partner's network, of the training of more than a thousand young communicators in favelas in three municipalities of Rio de Janeiro. Experiences of my personal path of care and transformation inspired my contributions in the Engajada*mente* Project.

**Rafaela de Oliveira da Cunha, Youth researcher**

As a 20-year-old young woman and student of Anthropology at a public university, I am in constant contact with everyday youth engagement and protagonism. I have always recognised the importance of youth participation, but it was only after being part of the project that I began to identify the power of action and of the voice of young people. Researching this allowed me to understand that many young people, despite being completely different individuals, go through similar situations on a daily basis, with similar emotions and thoughts that complement each other and could give voice to each other. It is noticeable how these young people identify with each other and, many times, they can get closer due to difficulties in taking a stand, asking for help and other factors that affect their lives, as well as their mental health and well-being. When they get together to fight for their rights, for example, they can often find a support network in this group, also based on their own experience, because they realize that they are not alone in a given situation, and this is even reinforced when adults are interested in the agenda brought by young people, as it happens in this research, it is even more motivating.

**Brenda Thallys Rocha Seabra, Youth researcher**

My adolescence was the period in which I most experienced radical changes in my development and in the way I related to the world. It was at this stage that potentialities were discovered as a black woman, as well as some vulnerabilities linked to social conditions and psychological vulnerabilities, not only mine but also those of many young people in my social circle. My personal experience was marked by common dissatisfactions and desires related to the Brazilian reality, involving social issues such as class, race and gender. Being an undergraduate student in Psychology was, and still is, the perfect place to respond to such concerns. Researching, talking about, studying and experiencing aspects related to mental health and youth protagonism within the Engajadamente Project is realising the relevance of my role in

contributing to individual and collective well-being by viewing young people's own responsibility as fuel for changes they want to see in their daily life.

**Julyana Alves Ferreira, Youth researcher**

My experience as a young person in Brazil has not been one of the easiest, even more so with so much violence historically perpetuated against the simple existence of the Brazilian people and the different realities that result from this, which are shaped by class, race, gender, among other factors. However, as a young person, I have an irremediable desire for change and as a Brazilian, an incessant desire to be heard. In this sense, being an undergraduate Psychology student and through participation in the Engajadamente Project, it was possible to give more meaning to what has always affected me within the position I occupy in the world. It was also important to stress the relevance of aspects regarding youth protagonism and engagement with mental health, in addition to enabling the recognition, despite the difficulties, of the enormous potential and voice that young people have within the different contexts in which they participate. The exchange of experiences, affections and daily sharing with other young people who share similar perspectives and with other different age groups was important to validate the young people's perspective on the world.

**Victor Hugo de Lima Santos, Youth researcher**

History has shown that several ruptures in society have come from youth dissatisfaction with their social reality. Through collective struggles, young people in Brazil and around the world have been building a prosperous future for future generations, based on what they believe is best for themselves and others. However, as a Brazilian teenager, full of social and emotional vulnerabilities, I had my power doubted in spaces where the voice of young people was seen as of lesser value. It was only in my experience as a co-researcher in the Engajada*mente* Project that I had the opportunity, for the very first time, to see myself as a social agent with the potential to promote change and lead actions that target youth mental health and inspire other young people to recognize their potential, regardless of the barriers and vulnerabilities that affect them.

**Rafa Ribeiro Alves de Souza, Youth researcher**

Young people's experiences towards overcoming barriers to support their peers' mental health deeply resonate with me. Throughout the research process, I was curious about the overall

sociopolitical awareness and bold determination displayed by young people due to the similarity between their experiences and mine. Moreover, the line between we young researchers and the other young people was thin sometimes. However, I recognise (or like to believe so) that the fluid and open process held during the interviews led to this semi-ethnographical relationship. One of the reasons I decided to study Political Science (as an undergraduate student) was to understand how power emerges within social structures and groups, and why some of them (such as LGBTQIAP+, black and young people's movements) struggle so much to achieve self-determination and the ability to solve their own problems. In the Engajadamente Project, the results confirmed what I always suspected to be the biggest of the barriers: young people are systematically not heard and that is why they shout even louder, at least I will.

**Gabriela Pavarini, Senior researcher**

As an immigrant woman in the UK and someone who grew up in Brazil, a country marked by historically rooted inequalities, inclusion and social participation have always been values I hold dear. As my research career progressed, I have discovered multiple ways to embed these values not only into themes of my research, but also the social relations of research production. Working with youth advisory groups has opened my eyes to the importance of placing adolescents at the centre of research and interventions about them. Since then, my research projects have increasingly involved and engaged adolescents across co-creation, research, and dissemination. This project was born of a desire to listen to Brazilian young people's *own* views on participation, during a time of great vulnerability. But it has also been an opportunity to explore more radical forms of youth involvement in research design and implementation. The close partnership between our senior research team and the Youth Collaborative Group has been of mutual capacity-building, and a huge catalyst for creativity and criticality. Nurturing intentional, co-constructive ways of knowing and being can be a powerful force for social change and community wellbeing.

# Supplementary material 4.

Initial codes (Phase II)

**Supplementary material 4.** Initial codes (Phase II)

# Initial codes - Phase II

| Name | Description | Files | References |
|---|---|---|---|
| Engagement and Aspirations | Young people's expressions, perceptions and ideas towards aspirations and engagement related to mental health promotion | 24 | 158 |
| Barriers to engagement & Peer Support | Issues, dynamics and structures that can impede or make it difficult to young people to employ their agency towards mental health promotion | 24 | 133 |
| Who is responsible for young people's mental health | Vignettes, examples and statements that indicate how young people understand who is responsible for supporting and promoting young people's mental health | 19 | 63 |
| Peer Support | Vignettes, examples and statements that indicate how young people promote peer support related to mental health issues | 16 | 59 |
| Mental health literacy | Vignettes, examples and statements that indicate young people's literacy in mental health | 17 | 33 |
| Space to be heard | Vignettes, examples and statements that indicate the lack of a legitimate and safe space in which young people can talk about mental health issues | 8 | 17 |
| Collective actions to promote mental health | Examples of actions that young people employ to promote mental health in a broad level | 7 | 12 |
| Partnership & Engagement | Vignettes, examples and statements that indicate partnerships young people have or wish to have in order to have a more active role in mental health promotion | 6 | 9 |
| Health promotion | Examples of actions that young people (wish to) engage in to promote mental health in general | 2 | 5 |

# Supplementary material 5.

Second level of analysis (Phase II)

**Supplementary material 5.** Second level of analysis (Phase II)

# Phase III – Second Level of Analysis: generating initial themes

**Number of candidate themes**: 4
**Number of features**: 8

## Candidate Theme 1: Aspirations Towards Mental Health Promotion

**General description**: this theme captures young people's aspirations towards the promotion of their peers' mental health and well-being. These aspirations regard their motivations to engage in direct peer support as well as actions to promote the well-being of their school.

### *Feature (a): Motivation*

***Description***: this feature captures young people's motivation to support their peers' mental health and well-being. Some feel a sense of agency and responsibility towards mental health promotions and others don't.
***Source:*** [P1, P2, P4, P5, P6, P9, P10, P11, P12, P13, P14, P15, P16, P18, P19, P20, P21, P24, P25, P26, P27, P31, P33, P34, P35, P37, P42, P43, P44, P45, P46]

### *Feature (b): Collective Action*

***Description***: young people's aspirations regarding collective actions to support their peers' mental health and wellbeing.
***Source:*** [P1, P2, P3, P4, P5, P6, P7, P8, P9, P10, P11, P13, P16, P17, P18, P19, P24, P25, P29, P31, P33, P35, P36, P42, P43, P44]

### *Feature (c): Peer Support*

***Description***: young people's aspirations regarding actions to support their peers' mental health and wellbeing.
***Source:*** [P1, P2, P3, P4, P6, P7, P8, P9, P10, P11, P12, P13, P14, P16, P17, P18, P19, P20, P21, P22, P23, P24, P26, P27, P28, P31, P32, P33, P34, P36, P38, P39, P40, P41, P42, P45, P46]

## Candidate Theme 2: Barriers to Employ their Agency

**General description**: this theme captures contextual issues that can impede or make it difficult for young people to employ their agency towards mental health promotion.

### *Feature (a): "They don't know much about life!": Underestimating Young People's Agency*

***Description***: this feature captures contextual issues that can lead to the underestimation of young people's potential for mental health promotion
***Source:*** [P1, P6, P,7, P13, P14, P15, P16, P17, P18, P19, P20, P21, P22, P28, P29, P33, P34, P35, P36, P42, P44, P45, P46]

### *Feature (b): The Lack of a Supportive Context*

**Supplementary material 5.** Second level of analysis (Phase II)

***Description***: this feature captures contextual factors that reflect a lack of support for young people's engagement in direct peer support as well as actions to promote the well-being of their school.
***Source:*** [P1, P5, P14, P16, P17, P19, P25, P26, P31, P37, P38, P40, P41, P42]

## Candidate Theme 3: Barriers to Deliver Peer Support

**General description**: this theme captures intrapersonal and interpersonal issues that can impede or make it difficult for young people to employ their agency towards mental health promotion.

### *Feature (a): "I want to help but I don't know how": lack of skills*

***Description***: this feature captures young people's lack of skills to promote mental health.
***Source:*** [P1, P3, P4, P5, P7, P9, P11, P15, P16, P17, P24, P25, P26, P27, P28, P30, P31, P44, P45, P46]

### *Feature (b): Self-efficacy*

***Description***: this feature captures young people's perceptions of their self-efficacy to support their peers' mental health and well-being.
***Source:*** [P2, P9, P13, P16, P17, P18, P20, P21, P22, P24, P25, P26, P27, P33]

### *Feature (c): Limits and Self-care*

***Description***: this feature captures young people's expressed limits for their engagement in mental health promotion as well as the need for self-care.
***Source:*** [P1, P2, P9, P10, P11, P12, P13, P14, P16, P17, P19, P24, P25, P26, P27, P28, P33, P34, P35, P41, P42, P43, P44, P45, P46]

## Candidate Theme 4: "*We Young People, We Understand Ourselves but We Need Other People*": Partnership and Liaising with Others to Employ Agency

**General description**: this theme captures the need to liaise with others or establish a partnership to properly employ their agency towards mental health promotion.

***Source:*** [P1, P2, P5, P11, P13, P18, P28, P32, P33, P34, P45, P46]

# Supplementary material 6.

Member reflections

**Supplementary material 7.** Member reflections protocol

# Reflecting on the research results

**Study title:** Young People's Sense of Agency and Responsibility towards Promoting Mental Health and Well-being

Dear young person,

Once again, we thank you for your interest and important contribution to the Engajada*mente Project*. With the information gathered from the focus groups and interviews that you took part in, we produced some results and we would like you to reflect on them with us. Before we begin, please fill in the following information:

| **PARTICIPANT INFORMATION** | |
|---|---|
| Age: | |
| State/City: | Race/ethnicity: |
| How do you define your gender? | |

Now, you will watch a short presentation of the results made by our Youth Collaborative Committee:

https://youtu.be/6827368TsOM

| **REFLECTIVE QUESTIONS** |
|---|
| ***Your Perception of the Results*** |
| After watching the presentation, what did you think of the results? |
| |

**Supplementary material 7.** Member reflections protocol

**Considering your experience as a young person and also your relationship with other young people, please answer the following questions:**

Our results showed that young people face several barriers to getting involved in mental health promotion actions at school. What did you think of these barriers?

| |
|---|
| |

Results indicated that young people have different ways of supporting and engaging in actions to promote mental health and well-being. What do you think of these different forms of action?

| |
|---|
| |

What did you think of the way the results addressed youth engagement in collective actions for the promotion of mental health and well-being?

| |
|---|
| |

What result was the most significant to you? Why?

| |
|---|
| |

What result did you think was the least significant? Why?

| |
|---|
| |

Were there any results that you would like to have seen more explored/discussed? Which one and why?

| |
|---|
| |

Overall, how relevant do you consider the results? Why?

| |
|---|
| |

**Supplementary material 7.** Member reflections protocol

| General Impressions about the Results | YES / NO |
|---|---|
| **Considering the results as a whole, please answer:** | |
| Do you think they were able to describe, in general, the involvement of young people in actions to promote mental health and well-being?<br>If 'No', please explain: | |
| Do you think they reflect your experience as a young person in a fair and representative way?<br>If 'No', please explain: | |
| Do you think they helped you to better understand the involvement of young people in actions to promote mental health and well-being?<br>If 'No', please explain: | |

# Supplementary material 7.

Compilation of participants' reflections

# Compilation of Participants' Reflections

We invited all 46 participants to take part in member reflections process. Ten of them agreed to take part and their sociodemographic information and accounts are presented below:

| **Age** | |
|---|---|
| Mean | 17.4 |
| Std. Deviation | 1.174 |
| Minimum | 16 |
| Maximum | 19 |

| **How do you define your gender?** | |
|---|---|
| | Frequency |
| Man (cis) | 1 |
| Gender fluid | 1 |
| Woman (trans) | 1 |
| Woman (cis) | 7 |
| Total | 10 |

As seen, most participants were women, and their mean age was 17.4. During the session, participants watched a video summarising the study's results and then they filled up a form asking open and closed question regarding the results and their reflections:

| **Do you believe the results were able to describe, in general, young people's engagement in mental health promotions and well-being actions?** | |
|---|---|
| | Frequency |
| Yes | 9 |
| No | 1 |
| Total | 10 |

| **Do you believe the results reflect your experience as a young person, in a fair and representative way?** | |
|---|---|
| | Frequency |
| Yes | 9 |
| No | 1 |
| Total | 10 |

| **Do you believe the results helped you better understand young people's engagement in mental health promotions and well-being actions?** | |
|---|---|
| | Frequency |
| Yes | 9 |
| No | 1 |
| Total | 10 |

**Supplementary material 7.** Compilation of participants' reflections

As seen, only one participant did not think the results were representative of young people's experiences in engaging in mental health promotion and well-being actions. In the open questions, this participant clarified that they liked the results as they were "very interesting, even though 46 [young people interviewed] is a very small number to generalise to all Brazilian young people". Below we present all participants' accounts in response to the open questions:

| Question | Answers |
|---|---|
| *After watching the video, what are your impressions regarding the results?* | "I liked it. When I started to play the game [*Cadê o Kauê?*], I found it very interesting" **(Participant 1)**<br><br>"Very elucidating. I loved it" **(Participant 2)**<br><br>"I found it very interesting, even though 46 [young people interviewed] is a very short number to generalise to all Brazilian young people" **(Participant 3)**<br><br>"Really cool, very important. They are essential to improve young people's mental health. It was also a great opportunity to give young people a voice" **(Participant 4)**<br><br>"I liked them and I agree with them" **(Participant 5)**<br><br>"Very good" **(Participant 6)**<br><br>"Very good. They fit what was researched with us young people and everything is well summarised and interpreted. I felt represented!" **(Participant 7)**<br><br>"I think they are good results. During the meetings [focus groups], all the different opinions and perspectives were clear, which is natural as everyone has their own way to think and their own reality" **(Participant 8)**<br><br>"I found the results extremely important and necessary to understand a little bit more about mental health, which is not a subject very discussed. They all help to understand the ways in which one can deal with mental health" **(Participant 9)**<br><br>"I believe the results are coherent with the reality and with the research" **(Participant 10)** |

**Supplementary material 7.** Compilation of participants' reflections

| | |
|---|---|
| *Our results show that young people can face some barriers to engage in mental health promotion and well-being actions, particularly in schools. What are your impression regarding these barriers?* | "Like people who do not care about others' mental health. Sometimes, we have to fight [for our mental health] by ourselves" **(Participant 1)**<br><br>"I understand but they could be smaller" **(Participant 2)**<br><br>"I believe they are mainly social and cultural barriers. They lead to difficulties in valuing mental health as well as having time and money needed to be involved in mental health promotion" **(Participant 3)**<br><br>"These barriers are very realistic and they match my own experience" **(Participant 4)**<br><br>"Sometimes, we are afraid of being invasive and also afraid of not having enough credibility [to engage in mental health promotion]" **(Participant 5)**<br><br>"I believe there is a lack of support at schools and where there is any support, students feel scared to engage. The action should start with teachers so that students would feel more comfortable [to engage]" **(Participant 6)**<br><br>"These are barriers we really see every day when we try to help some in need. We have different ways to address these sensitive subjects" **(Participant 7)**<br><br>"The most difficult barrier is the fact that some people still think that mental health problems are bullshit or that people who struggle are exaggerating, and therefore these issues should not be addressed or solved urgently" **(Participant 8)**<br><br>"I think these barriers are really very complicated and really prevent our involvement in actions towards mental health, it is very difficult to address these issues due to lack of opportunity and support" **(Participant 9)**<br><br>"The lgbtqia+ prejudice… for being trans, many people invalidate our opinions when dealing with serious matters due to our marginalisation" **(Participant 10)** |
| *The results indicate that young people have different ways of supporting and leading actions to promote mental health and well-being. What did you think of these different forms of action?* | "I like the family approach" **(Participant 1)**<br><br>"Interesting, being young is that… it is reinventing ourselves"**(Participant 2)**<br><br>"I thought it was cool to think of new forms of health promotion, mainly talking about those carried out in schools" **(Participant 3)**<br><br>"I found it essential, it shows how we young people are concerned about our health, and how this is an emergency for us" **(Participant 4)**<br><br>"I agree with the ways outlined; every person helps as they can and feel comfortable with" **(Participant 5)**<br><br>"Very good. The more ways, the better. Sometimes something that works for me won't work for someone else" **(Participant 6)**<br><br>"They are very relevant because we try to help in whatever way is best for the person" **(Participant 7)** |

**Supplementary material 7.** Compilation of participants' reflections

| | |
|---|---|
| | "These are differences from what I already thought they would be, because depending on the age and particular experiences of each one, the way of thinking and dealing with this subject would be different" **(Participant 8)**<br><br>"All of them are valid, and necessary, they can certainly help even if it's just a little" **(Participant 9)**<br><br>"I believe they focused on centralizing simple acts that make all the difference in everyday life" **(Participant 10)** |
| *Which result was the most significant for you? Why?* | "When they [young people] start to worry about each other" **(Participant 1)**<br><br>"young people help each other in any way they can" **(Participant 2)**<br><br>"Seeing the themes raised by young people, it is important that we raise our agenda" **(Participant 3)**<br><br>"For me, the most significant result was the diversity of the people who participated, because it is no use giving voice to just one group of young people, we need to give voice to different life experiences and different demands" **(Participant 4)**<br><br>"The result outlining that young people are interested in helping others. It's good to know that if they get a little encouragement, they will help more and more the next one" **(Participant 5)**<br><br>"I don't know" **(Participant 6)**<br><br>"The fact that young people feel responsible for the mental health of others, because that touches me and makes me think, it really is a great challenge to deal with the mental health of others, but I feel responsible for that, but it is also a huge responsibility for me" **(Participant 7)**<br><br>"Todos" **(Participant 8)**<br><br>"They were all very meaningful to me, I think I can learn a little something from each one of them and take it as a lesson" **(Participant 9)**<br><br>"A personal result, I say, is that after the project I began to notice the small actions that totally change the daily lives of the people around me and this has a great impact on the well-being of others and the social group" **(Participant 10)** |
| *Which result did you feel was least significant? Why?* | "None" **(Participant 1)**<br><br>"Young people find it difficult to engage in schools, this is obviously seen in our daily lives" **(Participant 2)**<br><br>"The number of young people is a small percentage compared to the number of regions and social subgroups present in each part of the country" **(Participant 3)**<br><br>"Hum, I don't know, I think they are all important, the least significant maybe is the way young people help their friends' mental health, because they are not always helping to solve the problem, sometimes we end up harming our own health because of a friend we want to help" **(Participant 4)**<br><br>"I didn't think it had a less significant result, but I couldn't hear the audio very well when they talked about responsibility... if I had to choose one, it would |

| | |
|---|---|
| | be this one" **(Participant 5)**<br><br>"I don't know"**(Participant 6)**<br><br>"None, all are essential and important" **(Participant 7)**<br><br>"All themes were very important to me, since I participated in almost all meetings" **(Participant 8)**<br><br>"None of them" **(Participant 9)**<br><br>"I believe that none, all results were very relevant and important due to the fact that all of them was very direct and objective in their messages, when we talk about well-being and mental health, we think of big actions, but it is much more about small daily actions instead of big moves" **(Participant 10)** |
| *Were there any outcomes that you wish had been further explored/discussed? If so, which one and why?* | "No"**(Participant 1)**<br><br>"I don't know"**(Participant 2)**<br><br>"No"**(Participant 3)**<br><br>"Yes, I think the difference in mental health between heterosexual people and LGBT young people should be explored, because LGBT young people are much more prone to mental health problems" **(Participant 4)**<br><br>"There was none" **(Participant 5)**<br><br>"I think they discussed a lot about mental health at school, but I would like them to talk more about within the family, what to do, how to seek help" **(Participant 6)**<br><br>"No" **(Participant 7)**<br><br>"No" **(Participant 8)**<br><br>"All seemed well explored to me" **(Participant 9)**<br><br>"With regard to groups led by young people, the support networks led by young people are still not very visible, these groups have a direct basis for the difference in youth, after all, they are young people talking to young people" **(Participant 10)** |
| *In general, how relevant do you consider the results of this research? Why?* | "Very important, because young people are lost and don't know what to do" **(Participant 1)**<br><br>"Very important! I've never seen anything like this before and I can't wait to share it with more people" **(Participant 2)**<br><br>"Very relevant, as it has given rise to a material in which I believe" **(Participant 3)**<br><br>"Extremely relevant, because these data will help create public policies on the subject" **(Participant 4)**<br><br>"I think it's very relevant because it shows that young people are interested in mental health" **(Participant 5)**<br><br>"Very relevant, the game will help many people" **(Participant 6)** |

**Supplementary material 7.** Compilation of participants' reflections

| | |
|---|---|
| | "A lot, because it will help the project and it will help us" **(Participant 7)**<br><br>"This research was very important and it was very nice to have participated in it. Because talking about mental health is very important, even more so in adolescence" **(Participant 8)**<br><br>"The results are good, it is always important to address the topic [of mental health] and results like these are of great help and impact" **(Participant 9)**<br><br>"Super relevant, showing people how young people feel about their health is extremely important for those outside the bubble of the new generation, after all, they need to understand that the new generation has an opinion and wants and needs to be heard" **(Participant 10)** |
