# Supplementary material for: Young people’s sense of agency and responsibility towards promoting mental health in Brazil: a reflexive thematic analysis
Source: BMJ Open. 2024 Dec 7;14(12):e084996. doi: 10.1136/bmjopen-2024-084996 (PMC11628997; doi:10.1136/bmjopen-2024-084996)
Supplement: Supplementary file 2 [file bmjopen-2024-084996.reviewer_comments.pdf]

PEER REVIEW HISTORY

BMJ Open publishes all reviews undertaken for accepted manuscripts. Reviewers are asked to complete a checklist review form and are provided with free text boxes to elaborate on their assessment. These free text comments are reproduced below.

ARTICLE DETAILS

Title (Provisional)

Young people's sense of agency and responsibility towards promoting mental health in Brazil: A reflexive thematic analysis

Authors

de Alcântara Mendes, Josimar Antônio; Murta, Sheila Giardini; Siston, Felipe Rodrigues; de Oliveira da Cunha, Rafaela; Seabra, Brenda Thallys Rocha; Alves Ferreira, Julyana; Ribeiro Alves de Souza, Rafa; de Lima Santos, Victor Hugo; Singh, Ilina; Pavarini, Gabriela

---

VERSION 1 - REVIEW

**Reviewer**	**1**
**Name**	**Sapiro, Beth**
**Affiliation**	**Montclair State University**
**Date**	**20-Mar-2024**
**COI**	**N/A**

Thank you for the opportunity to review this manuscript. It addresses an important topic and provides a valuable contribution to the research literature. More than that, it provides an impressive example of how to co-produce and disseminate research on youth mental health promotion in an innovative and accessible way. The supplemental materials provide a wealth of valuable resources for other researchers, especially the structured guide for reflexivity. I offer a few minor suggestions that could further strengthen the manuscript.

1. The background section of the paper is well written and makes a persuasive case for the study. It could be strengthened with a discussion about the particular political and cultural context of youth mental health in Brazil. Some of these details are mentioned in the discussion section; however, I think it would be helpful to introduce these earlier, such as in the introduction to the paper. What is useful for an international audience to know about the cultural values, political climate, stigma, and access to treatment that young people in Brazil currently navigate?

2. Minor typographical observation in the first paragraph (p. 5, line 31) - should probably be "rights-bearing"

3. In what language were the focus groups and interviews conducted? If transcripts were translated into English from Portuguese, it might be helpful to state how they were translated.

4. In the discussion of data analysis, I was not familiar with the phrase “ipsis litteris” (page 12, line 40). Perhaps include a definition or a few words of explanation.

5. The use of member reflection is important for establishing the trustworthiness and credibility of the findings. It would be helpful to include some detail about how these reflections were collected.

6. In the Results section, the section for the final theme (Theme 4) would benefit from a summarizing sentence or two at its conclusion, similar to the previous three themes.

7. The discussion section is excellent and has valuable implications for both researchers as well as adults looking to partner with young people in mental health promotion. One topic that could be further discussed in this section is the intersectionality of mental health stigma with other forms of oppression that young people encounter, including racism and anti-LGBTQ bias. It seems from the description of the participants that these forms of oppression may also have been part of their experience. If they were not explored in the current study, it would be a worthwhile topic for a future study.

Reviewer 2

Name Williams, Kofoworola

Affiliation Drexel University Dornsife School of Public Health, Community Health and Prevention

Date 02-Apr-2024

COI None to disclose.

Overall, the topic is significant and an important issue to draw attention to. However, the extent to which a reader outside of this scope would buy the significance is not certain. Mainly, the background seems a bit superfluous and includes information that can lead the reader to not grasp the significance fully. It is appreciated to read about participatory approaches but when it moves into discussions on agency and moral responsibility, it switches gears quick and does not appropriately lead to youth participation and peer support which arguably varies. Peer support could be flushed out a bit more since it's important to this paper. It is suggested that the background be revised to focus more pointedly on why we should care about this population and the outcome being studied within the context of peer support as a delivery and health promotion avenue and how incorporating elements of youth participation and agency furthers a researcher's ability to address related health inequities and stigma, even. And then it is not quite clear how this all

ties in to addressing the barriers listed on page 7. Overall, the background could be tighter and more concise. Additionally, it might be helpful to add more in to the limitations and future directions sections to drive home the significance, generally, and the contribution of this work to the field. And implications.

VERSION 1 - AUTHOR RESPONSE

Reviewer: 1 (Dr. Beth Sapiro, Montclair State University)

Reviewer: Thank you for the opportunity to review this manuscript. It addresses an important topic and provides a valuable contribution to the research literature. More than that, it provides an impressive example of how to co-produce and disseminate research on youth mental health promotion in an innovative and accessible way. The supplemental materials provide a wealth of valuable resources for other researchers, especially the structured guide for reflexivity. I offer a few minor suggestions that could further strengthen the manuscript.

Authors: Thank you very much for your kind feedback. We are very pleased to hear that you found the contribution valuable and the materials helpful for future work.

Reviewer: The background section of the paper is well written and makes a persuasive case for the study. It could be strengthened with a discussion about the particular political and cultural context of youth mental health in Brazil. Some of these details are mentioned in the discussion section; however, I think it would be helpful to introduce these earlier, such as in the introduction to the paper. What is useful for an international audience to know about the cultural values, political climate, stigma, and access to treatment that young people in Brazil currently navigate?

Author: Thank you for the feedback. We have edited our opening paragraph (page 5) such that it now predominantly discusses the Brazilian context, including prevalence of youth mental health challenges, stigma, and access to treatment. We have also moved the sentence in the Discussion that talks threats to children’s rights under recent political climate onto the Introduction (page 7).

Reviewer: Minor typographical observation in the first paragraph (p. 5, line 31) - should probably be “rights-bearing”

Authors: We have corrected that. Thank you!

Reviewer: In what language were the focus groups and interviews conducted? If transcripts were translated into English from Portuguese, it might be helpful to state how they were translated.

Authors: Thank you for raising this helpful point. We have clarified that 'both focus groups and interviews were conducted in Portuguese' on page 11, and that 'The data analysis was conducted using the original Portuguese-language data. Therefore, only the excerpts presented in this study have been translated.'

Reviewer: In the discussion of data analysis, I was not familiar with the phrase "ipsis litteris" (page 12, line 40). Perhaps include a definition or a few words of explanation.

Authors: Thank you. We have replaced the term by 'verbatim'.

Reviewer: The use of member reflection is important for establishing the trustworthiness and credibility of the findings. It would be helpful to include some detail about how these reflections were collected.

Authors: On page 14, we have clarified how the reflections were collected:

'Participants were recontacted via email or text and sent an open-ended survey. The survey began with a video summary of the results produced by the YCG. Following the video, participants were asked questions to reflect on the relevance and significance of the results, to consider whether the results accurately represented their views, and to provide their own interpretations of the findings'.

Reviewer: In the Results section, the section for the final theme (Theme 4) would benefit from a summarizing sentence or two at its conclusion, similar to the previous three themes.

Authors: Thank you for the suggestion. We have added the following summarising sentence:

'In sum, this theme illustrates the complex intrapersonal factors — including barriers of self-doubt, lack of skills and fear of causing harm, but also respect for one's own needs — that underlie young people's choices on whether to offer support.'

Reviewer: The discussion section is excellent and has valuable implications for both researchers as well as adults looking to partner with young people in mental health promotion. One topic that could be further discussed in this section is the intersectionality of mental health stigma with other forms of oppression that young people encounter, including racism and anti-LGBTQ bias. It seems from the description of the participants that these forms of oppression may also have been part of their experience. If they were not explored in the current study, it would be a worthwhile topic for a future study.

Authors: Thank you for the helpful suggestion. On page 26 where we talk about stigma-reducing policies we added that "These policies must also consider the intersectionality of mental health stigma with other forms of oppression that young people encounter, including racism and anti-LGBTQ+ bias, which were part of the experiences of young people reported in the current study." In future directions we also highlighted the need for future studies using an intersectional lens in data analysis.

Reviewer 2 (Dr. Kofoworola Williams, Drexel University Dornsife School of Public Health)

Comments to the Author:

Overall, the topic is significant and an important issue to draw attention to.

However, the extent to which a reader outside of this scope would buy the significance is not certain. Mainly, the background seems a bit superfluous and includes information that can lead the reader to not grasp the significance fully.

It is appreciated to read about participatory approaches but when it moves into discussions on agency and moral responsibility, it switches gears quick and does not appropriately lead to youth participation and peer support which arguably varies. Peer support could be flushed out a bit more since it's important to this paper.

It is suggested that the background be revised to focus more pointedly on why we should care about this population and the outcome being studied within the context of peer support as a delivery and health promotion avenue and how incorporating elements of youth participation and agency furthers a researcher's ability to address related health inequities and stigma, even.

And then it is not quite clear how this all ties in to addressing the barriers listed on page 7. Overall, the background could be tighter and more concise.

Authors: Many thanks for these helpful comment. We have majorly revised our introduction. We have included an opening paragraph that discusses the Brazilian context; added more information on peer support the benefits related to equity and stigma reduction; added a better link to the section on agency/responsibility and the barriers listed on page 7; and added a stronger justification to the study at the end of the introduction. We have also shortened the text excluding any information that might be considered superfluous, making it tighter and more precise. We hope these changes meet the reviewer's expectations.

Reviewers: Additionally, it might be helpful to add more in to the limitations and future directions sections to drive home the significance, generally, and the contribution of this work to the field. And implications.

Authors: Thank you for the suggestion. We have revised the limitations section to include 'strengths' of the study, highlighting the co-produced element. We have also revised our conclusions section such that the significance, main contribution, and main implication of the study are more clearly stated.

VERSION 2 - REVIEW

**Reviewer**	**1**
**Name**	**Sapiro, Beth**
**Affiliation**	**Montclair State University**
**Date**	**04-Nov-2024**
**COI**	

The revisions are excellent and further strengthen the manuscript. No additional changes requested. Recommend accept.
